# Statistical Evaluation of Transformation Methods Accuracy on Derived Pathological Vectorcardiographic Leads

**DOI:** 10.1109/JTEHM.2022.3167009

**Published:** 2022-04-13

**Authors:** Jaroslav Vondrak, Marek Penhakert

**Affiliations:** Faculty of Electrical Engineering and Computer ScienceVSB-Technical University of Ostrava 708 33 Ostrava Czech Republic

**Keywords:** Transformation methods, transformation matrix, vectorcardiography, statistical evaluation

## Abstract

**Objective:** Vectorcardiography (VCG) as an alternative form of ECG provides important spatial information about the electrical activity of the heart. It achieves higher sensitivity in the detection of some pathologies such as myocardial infarction, ischemia and hypertrophy. However, vectorcardiography is not commonly measured in clinical practice, and for this reason mathematical transformations have been developed to obtain derived VCG leads, which in application in current systems and subsequent analysis can contribute to early diagnosis and obtaining other useful information about the electrical activity of the heart. **Methods and procedures:** The most frequently used transformation methods are compared, namely the Kors regression method, the Inverse Dower transformation, QLSV and the Quasi-orthogonal transformation. These transformation methods were used on 30 randomly selected records with a diagnosis of myocardial infarction from the Physikalisch-Technische Bundesanstalt (PTB) database and their accuracy was evaluated based on the calculation of the mean square error (MSE). MSE was subjected to statistical evaluation at a significance level of 0.05. **Results:** Based on statistical testing using the nonparametric multiselective Kruskall-Wallis test and subsequent post-hoc analysis using the Dunn method, the Kors regression as a whole method achieved the most accurate transformation. **Conclusion:** The results of statistical analysis provide an evaluation of the accuracy of several transformation methods for deriving orthogonal leads, for possible application in measuring and evaluation systems, which may contribute to the correct choice of method for subsequent analysis of electrical activity of the heart at orthogonal leads to predict various diseases.

## I. Introduction

Electrocardiogram (ECG) is currently the most common method in clinical practice for the diagnosis of heart disease. The ECG is most often measured as the potential difference between the electrodes placed on the patient’s chest. The most commonly used clinical ECG system is the 12-lead ECG, which monitors basic cardiac electrical activity from 12 different angles.

Another method that measure the electrical activity of the heart is vectorcardiography (VCG), which represents a slightly different approach. The basic mathematical model describing the heart cycle as a moving dipole. This dipole can be represented as vector of the electrical forces of individual heart cells [1]. This vector is represented by three orthogonal components and time, where direct measurement of these components is the basis of vectorcardiography.

The VCG is projected into three mutually perpendicular planes: sagittal, transversal and frontal and is most often measured using Frank lead system [1]. Other VCG lead systems that have been published are for example McFee and Parungao [2], SVEC III [3], and hybrid lead systems [4]. However, these systems were used rarely. Nowadays, the visualization of a vectorcardiogram is usually performed using specialized software. The curve of the vectorcardiogram has the shape of a curve determined by the time-traveling vector of the dipole moment. This method has proved promising because it shows changes in the direction and magnitude of the electrical forces of the heart revolution. This information is very important for the diagnosis of acute coronary syndromes, unfortunately, classical methods such as ECG are still preferred in clinical practice [5]–[7]. The benefits of a 12-lead ECG for the diagnosis of heart disease are unquestionable. However, there are studies whose results indicate a higher sensitivity of VCG, for example in the diagnosis of atrial enlargement and right ventricular hypertrophy, and it has been suggested to reconsider the frequency of 12-lead ECG use in favor of VCG in clinical practice [8], [9]. The use of VCG has also become useful in some specific situations, such as the assessment of intraventricular conduction defects in combination with inactive areas, identification and localization of ventricular preexcitation, evaluation of specific aspects of Brugg syndrome and estimation of the severity of some enlargements [10]. VCG also achieves higher sensitivity in the QRS complex analysis for better patient selection for cardiac resynchronization therapy, detection of myocardial injury [11] or extraction of VCG features from QRS [12] to detect ischemia.

**FIGURE 1.**
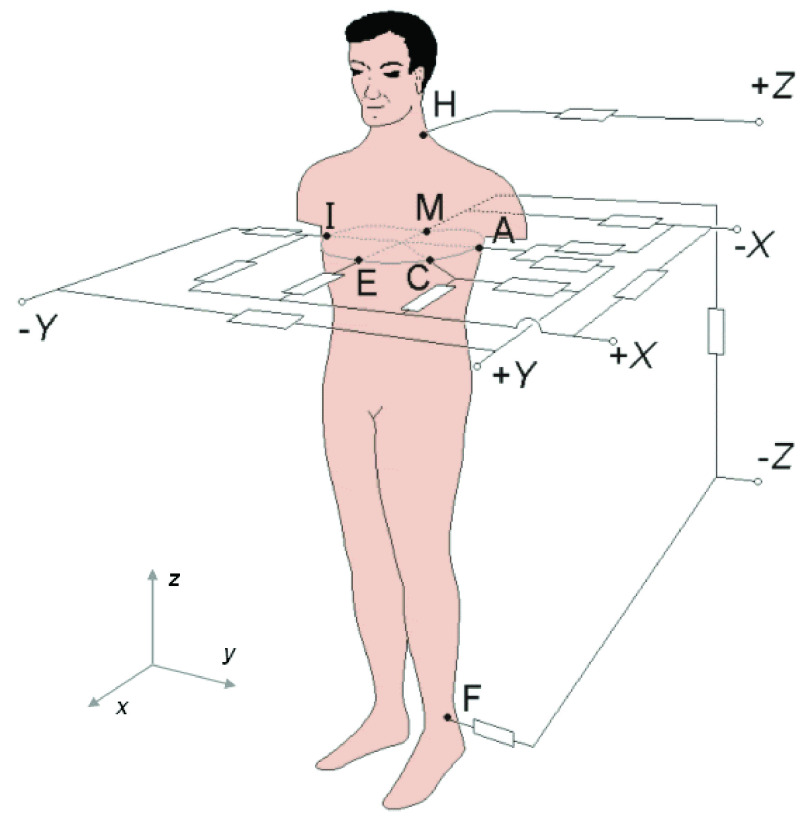
Location of electrodes in Frank lead system [13].

Vectorcardiography can be measured by several different lead systems, but Frank orthogonal lead system is the most common in clinical practice. It is measured using three bipolar leads that are perpendicular to each other. It consists of seven electrodes marked with capital letters I, E, C, A, M, F and H. Each electrode has its location, see the Figure 1.

Electrode H is located on the neck, electrode F on the left leg. Electrode E is located on the front of the chest and electrode M is located opposite on the back. Electrode I is located on the right central axillary line and electrode A on the left axillary line. The last electrode C is placed between the electrodes A and E due to the closest position to the heart [14]. As can be seen in Figure 1, the individual electrodes are routed through a resistor network. Malmivuo and Plonsey [13] explains the correct connection of these resistors and their correct values in their book. The individual VCG leads are derived using mathematical equations 1, where $P_{x}$, $P_{y}$ and $P_{z}$ represents the potentials at the individual electrode clips and I, E, C, A, M, F, H represent the individual electrodes [14]. Macfarlane *et al.* [15] explained the calculations of equations 1, which are based on a resistor network.

\begin{align*} P_{x}=&0,610\cdot A+0,171\cdot C-0,781\cdot I \\ P_{y}=&0,655\cdot F+0,345\cdot M-1,000\cdot H \\ P_{z}=&0,133\cdot A+0,736\cdot M-0,264\cdot I-0,374\cdot E \\&-\,0,231\cdot C\tag{1}\end{align*}

Despite some advantages over standard methods, VCG is rarely measured in clinical practice. The main reason is the inexperience of this method among healthcare professionals and also the insufficient computer technology of the last century, where the first loops were drawn by hand on graph paper. Due to the lack of VCG recordings and well established 12-lead ECG, transformation methods for converting 12-lead ECGs to VCG leads were derived. These methods could then be used in the systems to obtain additional important information. The accuracy of the transformation method is key in obtaining an accurate derived VCG record, especially in pathological records, where transformation achieves worst accuracy. In the following chapters, attention is paid to the most frequently used transformation methods in publications for the conversion of 12-lead ECG to 3-lead VCG.

## II. Transformation Methods

The first consideration of the possible transformation of individual lead systems was presented by the authors in [16]. In publication [17], Kornreich described that 12-lead ECG and Frank lead system are very similar in terms of their information content. Therefore, he stated that their mutual transformation is possible. This was followed by the first attempts at transformation based on transformation from VCG to 12-lead ECG by Dower *et al.* [18], [19]. Later, Wolf *et al.* [20] pointed out, that the measurements from orthogonal leads can contribute to better diagnostics. He therefore considered the possibility of creating a bidirectional method for the transformation of 12-lead ECG and VCG. The focus of transformation methods is mainly on QRS complex, however, there are also transformations that focus on other parts of the cardiac cycle. The mathematical notation of the transformation method can be expressed as a multiplication of two matrices according to equation 2, where $VCG_{der}$ represent transformed VCG leads, $M$ is transformation matrix and $E$ is matrix whose rows are formed by independent ECG leads.

\begin{equation*} VCG_{der} = M \cdot E\tag{2}\end{equation*}

Transformation methods for deriving orthogonal lead systems are an important method for obtaining other useful information about cardiac activity.

### A. KORS Regression Transformation

The transformation matrix presented by Kors was based on a regression technique for a group of patients from the CSE (Common Standards for Electrocardiography) database. The coefficients of the transformation matrix were derived by minimizing the mean error between the directly measured VCG and the transformed VCG. In this way, Kors derived several transformation matrices for different ECG segments. He then found that the matrices did not differ much from each other. The resulting matrix, which is shown in Table 1, is based on the regression of the QRS complex [21].

**TABLE 1:** Transformation Coefficients of Kors Regression Method

Lead	I	II	V1	V2	V3	V4	V5	V6
X	0,38	−0,07	−0,13	0,05	−0,01	0,14	0,06	0,54
Y	−0,07	0,93	0,06	−0,02	−0,05	0,06	−0,17	0,13
Z	0,11	−0,23	−0,43	−0,06	−0,14	−0,20	−0,11	0,31

### B. Inverse Dower Transformation (IDT)

Dower *et al.* [18] introduced the possibility of obtaining 12-lead ECG from Frank lead system, from which he subsequently derived the transformation matrix [19], [22]. Later, Edenbrandt and Pahlm [23] derived a pseudo inverse matrix for deriving VCG from ECG leads. This matrix was named as the Inverse Dower transform whose coefficients are in Table 2.

**TABLE 2:** Transformation Matrix for Inverse Dower Transformation

Lead	I	II	V1	V2	V3	V4	V5	V6
X	0.156	−0.010	−0.172	−0.074	0.122	0.231	0.239	0.194
Y	−0.227	0.887	0.057	−0.019	−0.106	−0.022	0.041	0.048
Z	0.022	0.102	−0.229	−0.310	−0.246	−0.063	0.055	0.108

### C. PLSV and QLSV Transformation

The transformation matrices derived by the regression method focus mainly on the QRS complex and the accuracy of the transformation for the P and T waves is considered sufficient. Due to the fact that some pathologies also interfere to a significant extent in the P and T waves, it is necessary to have transformations focusing on these parts of the heart revolution. Therefore Guillem *et al.* [24] presented a transformation matrix based on a regression method that is optimized for the P wave called PLSV (P Least Square Value), whose coefficients are in Table 3. They also derived a matrix focused only on the QRS complex called QLSV (Q Least Square Value), see Table 4. Both of these matrices were derived from dataset of 124 patients.

**TABLE 3:** QLSV Transformation Matrix

Lead	I	II	V1	V2	V3	V4	V5	V6
X	0.199	−0.018	−0.147	−0.058	0.037	0.139	0.232	0.226
Y	−0.164	0.503	0.023	−0.085	−0.003	0.033	0.060	0.104
Z	0.085	−0.130	−0.184	−0.163	−0.193	−0.119	−0.023	0.043

**TABLE 4:** PLSV Transformation Matrix

Lead	I	II	V1	V2	V3	V4	V5	V6
X	0.370	−0.154	−0.266	0.027	0.065	0.131	0.203	0.220
Y	−0.131	0.717	0.088	−0.088	0.003	0.042	0.048	0.067
Z	0.184	−0.114	−0.319	−0.198	−0.167	−0.099	−0.009	0.060

### D. Quasi – Orthogonal Transformation

Recording from any ECG system can be converted to a vectorcardiographic loops but it cannot be considered a full-fledged orthogonal lead. These derived systems are called as quasi-orthogonal systems and are an approximation of Frank lead system. One of the most commonly used quasi-orthogonal systems is derived by Kors [22], whose expression can be represented according to equation 3.

\begin{align*} X=&V6 \\ Y=&II \\ Z=&-0,5 \cdot V2\tag{3}\end{align*}

Based on the measurement of the average absolute deviation and also from the evaluation of cardiologists, Kors concluded that the transformation method based on the regression approach achieves higher accuracy [21].

## III. Material and Methods

For the possible application of transformation methods to health care systems, sufficient analysis is needed to verify the significance of the individual method. Statistical analysis is performed to determine which transformation method achieves the highest accuracy in deriving orthogonal lead systems. The basis for statistical analysis is a set of sufficient data. These files must first be processed in an appropriate manner in order to be able to work with this data.

### A. Study Population

In this study, ECG records from the Physikalisch - Technische Bundesanstalt diagnostic database, which were recorded from healthy volunteers and patients with various heart diseases at the Cardiology Department of the Benjamin Franklin University Clinic in Berlin, Germany, were used. The individual records of this database consist of 15 simultaneously recorded signals: a conventional 12-lead ECG and 3-lead Frank orthogonal system. The signals were acquired for 2 minutes with a 16-bit resolution in the range of ±16.384 mV and sampled at a sampling frequency of 1 kHz. The PTB database contains records from 294 subjects with different diagnoses. For the purposes of this study, 30 records diagnosed with myocardial infarction were selected from this database [25], [26].

### B. Data Preprocessing

As these are biological data (ECG/VCG), interfering components are also measured together with the required signal. Most of the interfering signal components have already been removed from the original database, but there are still fluctuations on the isoelectric line. Before the statistical evaluation itself, it is necessary to remove baseline wandering. This was achieved by applying a Savitzky - Golay filter with a window length of 1200, which detects this unwanted interfering component in the signal. The window length value was based on the sampling frequency and signal length. The entire length of the record, which is two minutes, was used for preprocessing and subsequent analysis. For clarity, Figure 3 shows the portion of the record where the red curve indicates the filtered record and the blue curve indicates the original with marked fluctuating component (black).

**FIGURE 2.**
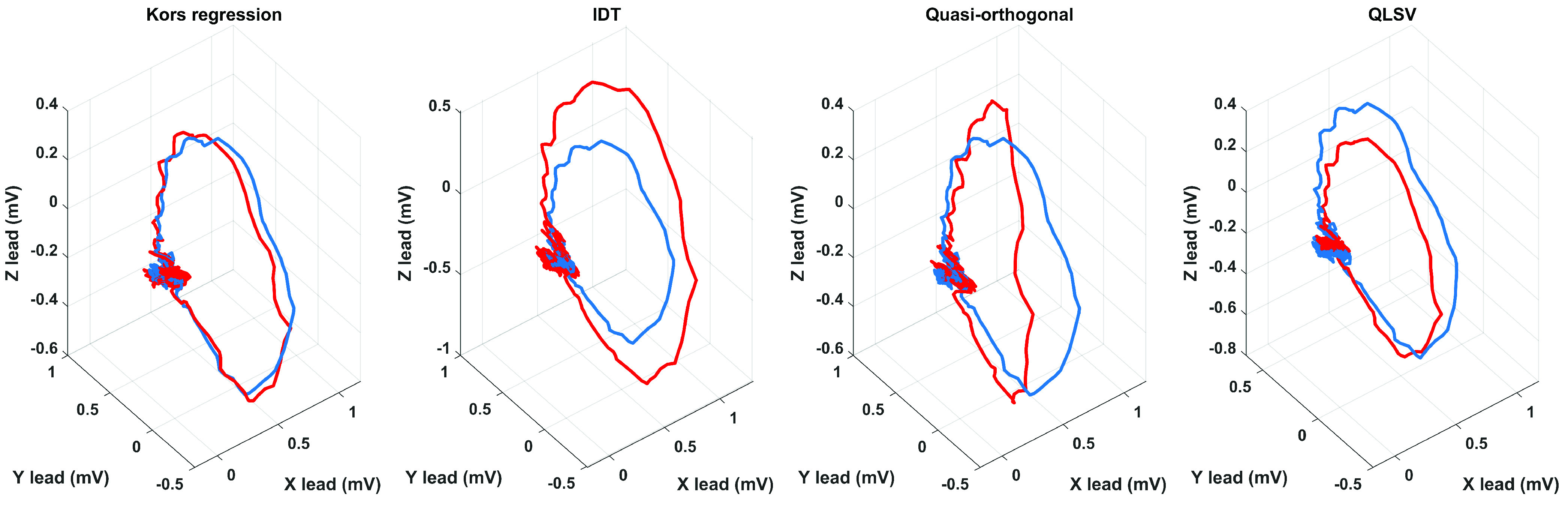
Examples of transformation methods used on a 12-lead ECG from a randomly selected record $s0554_{rem}$ with a diagnosis of myocardial infarction. The blue curve indicates the VCG measured by the Frank lead system and the red curve indicates the transformed curve.

**FIGURE 3.**
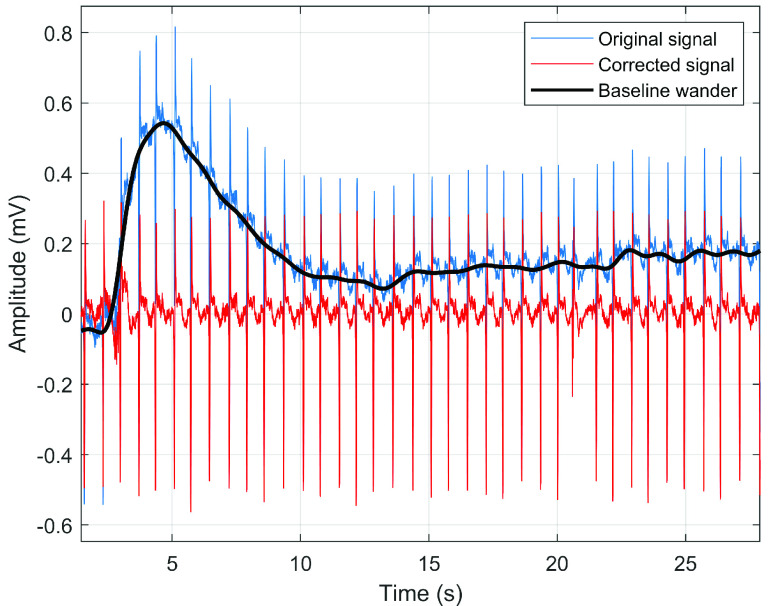
Filtration of baseline wandering from a randomly selected record $s0554_{rem}$ (lead Z) with a diagnosis of myocardial infarction.

After data alignment, linear transformation methods were applied to 12-lead ECGs to obtain derived orthogonal lead systems. These derived orthogonal lead systems were then compared with directly measured orthogonal leads measured by Frank lead system, which were considered here as reference values.

### C. Statistical Analysis

When testing hypotheses, the alpha significance level is determined. 95% (0.95) is considered a reliable estimate and the significance level is the remaining 5% (0.05). If the differences between the two files tested are less than alpha, then the records differ in significance level. In statistics, the mean and median are most often determined to perform a statistical test, then the statistical analysis is based on hypothesis testing. The null hypothesis ( $H_{0}$) represents equilibrium while the alternative hypothesis ( $H_{A}$) then expresses imbalance. Based on the selected statistical test, either the null hypothesis is rejected and the alternative hypothesis is accepted, or vice versa [27], [28].

Statistical analysis and all necessary calculations for individual methods were performed in the software environment Matlab 2018b. Statistical analysis was performed from the mean square error (MSE) results between the derived orthogonal system with the directly measured orthogonal system for each lead (X, Y, Z) calculated by equation 4.

\begin{equation*} MSE = \frac {1}{n}\cdot \sum _{i=1}^{n}\left ({V_{i}-oV_{i} }\right)^{2}\tag{4}\end{equation*}

where ${n}$ is the length of the record, *Vi* is the original measured vectorcardiography and *oVi* is the derived vectorcardiographic record. With this step, we got input values for statistical analysis.

In order to choose the right method enabling the comparison of MSE for individual methods and individual leads, it was necessary to verify the normality of the data. We tested the normality using the Shapiro - Wilk’s test. Shapiro - Wilk’s test, sometimes also known as W-test, can be defined as:

\begin{equation*} W=\frac {\left ({\sum a_{i}y_{i} }\right)^{2}}{\sum \left ({y_{i} - \bar {y} }\right)^{2}}\tag{5}\end{equation*}

where ${y}$ is an ordered sample of size ${n}$ to be tested, $a=(a_{1},\ldots,a_{n})^{\tau }$ is such that $\left ({n-1 }\right)^{-\frac {1}{2}}\sum a_{i}y_{i}$ is the best linear unbiased estimate of the standard of the $y_{i}$ assuming normality. From a practical point of view, a normal probability graph should always be accompanied by a W-test to provide qualitative information on the shape of the sample distribution [29], [30].

All tests are evaluated at a significance level of 0.05. In this case, they are multi-sample independent data and specific multi-sample tests of statistical analysis must be applied to them. These tests include, for example, the homoskedasticity tests of the Bartlett test, where the presumption of normality must be met, and the Levene test, which is less sensitive to the violation of the presumption of normality compared to the Bartlett test. We also have tests to verify the compliance of the mean values, respectively medians. These tests include ANOVA, where the assumption of normality and homoskedasticity must be met, the Kruskall-Wallis test, which is applied in case of non-compliance with normality.

As these are biological ECG/VCG data, where a normal distribution is usually not expected, non-parametric tests can be used. The Kruskal-Wallis test is a nonparametric analog of one-way analysis of variance, so it is sometimes called a nonparametric ANOVA. It is used when we want to compare the mean values of more than two independent sets based on a selection that does not meet the requirements for the use of parametric analysis of variance (especially normality). The procedure analogous to the Mann-Whitney test is used to calculate the observed value of the test statistic. It can be said that the Kruskal-Wallis test is an extension of the Mann-Whitney test to more than 2 selections. All ${n}$ observed values of the quantity are arranged in an increasing sequence and their order is determined. We arrange these orders in a table and determine the so-called sums of orders for individual $T_{n}$ selections. The total sum of all orders is (6). The (7) are used as test statistics [31], [32].

\begin{align*} T_{1}+\cdots +T_{n}=&\frac {n\left ({n+1 }\right)}{2} \tag{6}\\ Q=&-3\left ({n+1 }\right)+\frac {12}{n\left ({n+1 }\right)}\sum _{i=1}^{k}\frac {T_{i}^{2}}{n_{i}} \\&-\,3\left ({n+1 }\right)\tag{7}\end{align*}

The critical values of this statistic are tabulated in special tables. If the ranges of the individual selections are at least 5 elements, the test statistic ${Q}$ has approximately $X^{2}$ distributions with *k-1* degrees of freedom if the null hypothesis is valid. Then $p-value = 1-F_{0}$ ( $x_{OBS}$), where $F_{0}$ is a distribution function $X^{2}$ with *k-1* degrees of freedom and $x_{OBS}$ is observed value [31]–[33].

## IV. Results

**FIGURE 4.**
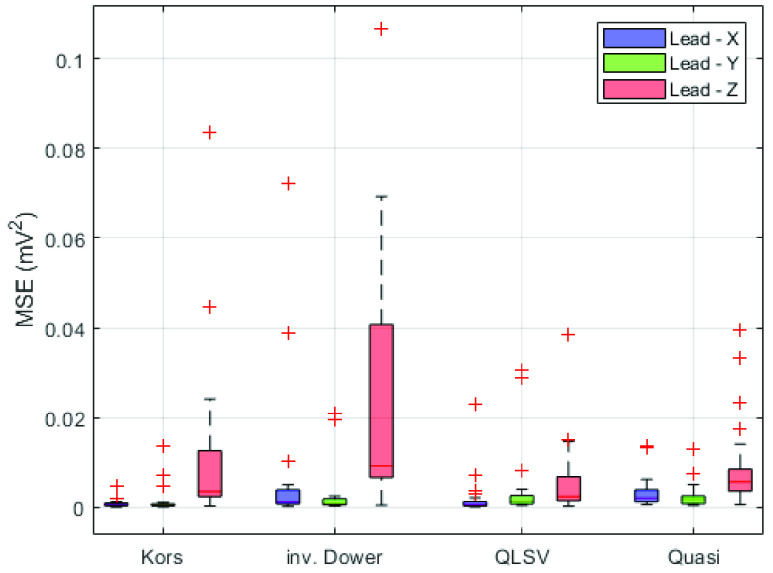
MSE for tested transformation methods in individual leads.

After applying the transformation method to the filtered 12-lead ECG data, the MSEs between the derived and directly measured orthogonal lead were calculated. Figure 4 shows boxplot visualization of the mean square error for all transformation methods used in individual leads.

Prior to the statistical analysis itself, remote observations were detected in the data by the method of internal walls. Due to the fact that these are biological data that no longer carry significant interfering components and values in remote observations can be expected, remote observations have been retained in the data set.

### A. Normality Test

To verify the normality of the data by Shapiro - Wilk’s test, the following hypotheses were tested:

•
$H_{0}$: Data is a selection from a normal distribution.
•
$H_{A}$: $\lnot H_{0}$ (negation of the null hypothesis)

**FIGURE 5.**
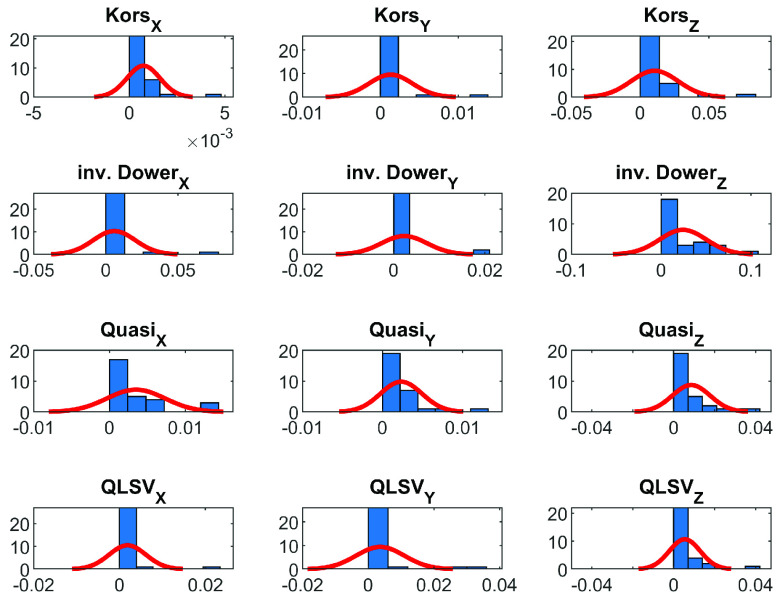
Gaussian curve with histograms of the tested methods.

All p-values are < 0.05. From the results of the p-values and based on Figure 5 it can be stated that the assumption about the normality of MSE must be rejected for all analyzed data and in further testing we move to non-parametric tests.

Another way to verify the normality of the data is to use a QQ diagram.

### B. Comparison of MSE for Compared Transformation Methods

Based on the results of the Shapiro – Wilk’s test, the data are subjected to the Kruskal - Wallis test. This test is a nonparametric analog to one-way analysis of variance, so it is sometimes called a nonparametric ANOVA. Just as analysis of variance is a multi-sample test of the agreement of the mean values, the Kruskal-Wallis test is a multi-sample test of the median agreement, generally the distribution agreement.

•
$H_{0}$: $x_{0,5}^{Kors}$ = $x_{0,5}^{IDT}$ = $x_{0,5}^{Quasi}$ = $x_{0,5}^{QLSV}$ (There are no statistically significant differences between the median MSE of the compared methods)
•
$H_{A}$: $\lnot H_{0}$ (At least one pair of methods differs statistically significantly in their median MSE)

From the Table 5 we can see that based on the Kruskal - Wallis test it is possible to reject the null hypothesis for all leads (p-value < 0.05), i.e. for all leads there is a statistically significant difference in the medians of MSE of the compared transformation methods. In the next step, it was determined by post-hoc analysis (multiple comparisons) whether there are so-called homogeneous groups of methods among the compared methods, which would give outputs with comparable MSEs on individual leads. We used Dunn’s method for post-hoc analysis. The results of the post-hoc analysis can be seen in the summary Table 6. The resulting order of transformation methods derived from the letter scheme can be seen in Table 7.

**TABLE 5:** Kruskal-Wallis Median Concordance Test of MSE for Individual Leads

Lead	X	Y	Z
p-value	< 0,001	< 0,001	< 0,001

**TABLE 6:** Results of the Post-Hoc Analysis of the Dunn Test for Each Lead

Lead X						
Dunn test (p-value)	Tr. method	Kors	Dower	Quasi	QLSV	
	Kors	*	< 0,001	< 0,001	0,905	
	Dower	*	*	0,828	0,020	
	Quasi	*	*	*	0,003	
	QLSV	*	*	*	*	
Post hoc analysis	Mean value ( $mV^{2}$)	Letter scheme				
	0,0007	Kors	A	*	*	*
	0,0019	QLSV	A	B	*	*
	0,0035	Kvazi	*	*	C	*
	0,0059	Dower	*	*	C	D
Lead Y						
Dunn test (p-value)	Tr. method	Kors	Dower	Quasi	QLSV	
	Kors	*	0,210	< 0,001	< 0,001	
	Dower	*	*	0,136	0,267	
	Quasi	*	*	*	0,999	
	QLSV	*	*	*	*	
Post hoc analysis	Mean value ( $mV^{2}$)	Letter scheme				
	0,0013	Kors	A	*	*	*
	0,0023	QLSV	A	B	*	*
	0,0023	Kvazi	*	B	C	*
	0,0036	Dower	*	B	C	D
Lead Z						
Dunn test (p-value)	Tr. method	Kors	Dower	Quasi	QLSV	
	Kors	*	0,002	0,990	0,740	
	Dower	*	*	0,014	< 0,001	
	Quasi	*	*	*	0,319	
	QLSV	*	*	*	*	
Post hoc analysis	Mean value ( $mV^{2}$)	Letter scheme				
	0,0054	Kors	A	*	*	*
	0,0086	QLSV	A	B	*	*
	0,0105	Quasi	A	B	C	*
	0,0242	Dower	*	*	*	D

**TABLE 7:** Resulting Order for Each Lead From Post-Hoc Analysis

Resulting order for lead X		Resulting order for lead Y		Resulting order for lead Z	
1.	Kors, QLSV	1.	Kors	1.	QLSV, Kors, Quasi
2.	Quasi, Dower	2.	Dower, Quasi, QLSV	2.	Dower

Based on the results of the post-hoc analysis in lead X, it can be argued at the significance level of 0.05 that the MSEs of the Kors regression method and the QLSV method is statistically significantly lower compared to the MSEs of the Quasi-orthogonal method and IDT.

The results of the post-hoc analysis in the Y lead, it can be argued at the significance level of 0.05 that the MSEs of the Kors regression method is significantly lower than for the other transformation methods.

**FIGURE 6.**
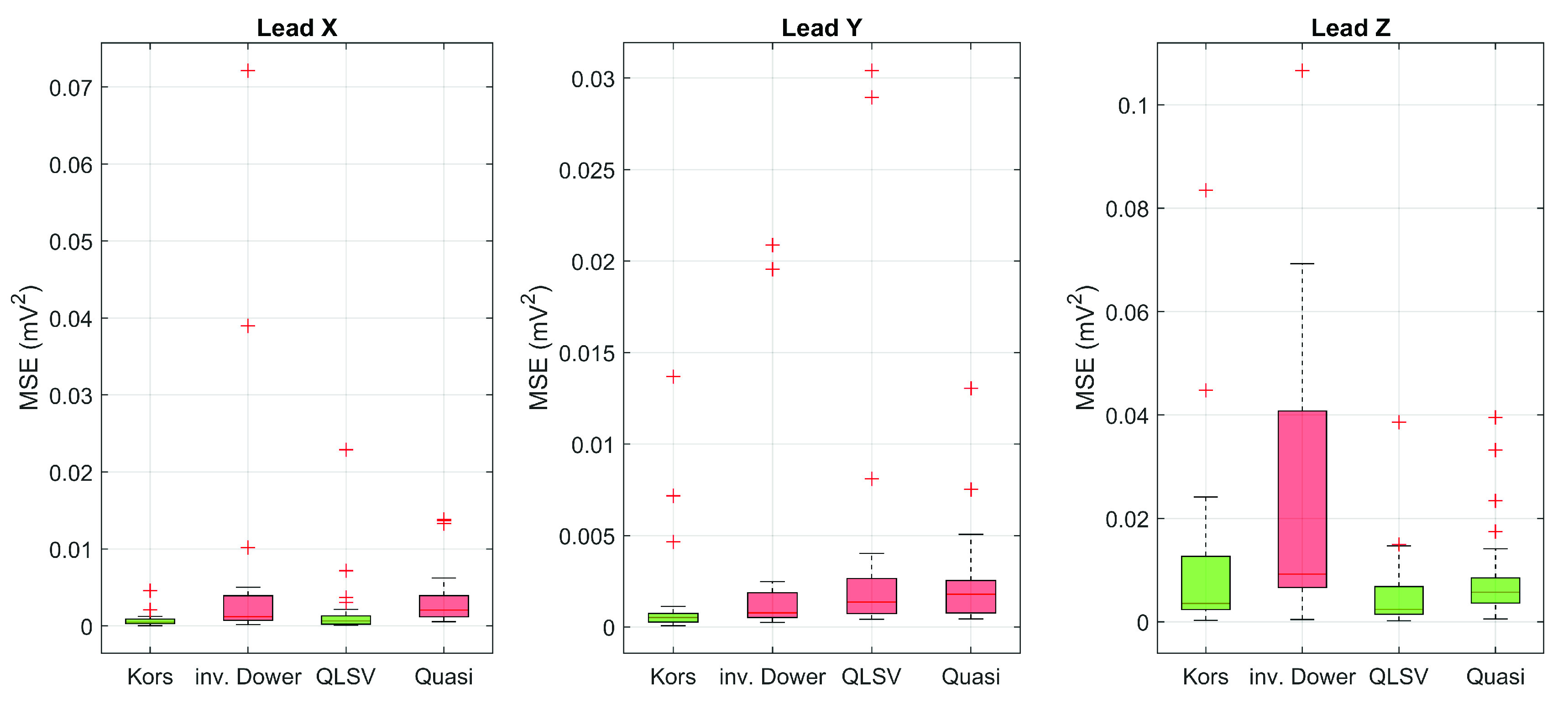
Graphical representation of post-hoc analysis for individual leads.

In the Z lead, it can be argued at the significance level of 0.05 that the MSEs of the QLSV, Quasi-orthogonal and Kors regression methods are significantly lower than in IDT. A graphical representation of the post-hoc analysis can be seen in Figure 6.

## V. Discussion

Transformation methods were derived to obtain a derived orthogonal lead system from a 12 lead ECG to obtain additional information from electrical activity of the heart. There are many transformation methods. The most frequently used transformation methods include the Kors regression method, IDT, QLSV and the Quasi-orthogonal method. Examples of the transformation methods used in this work can be seen in Fig. 2, where the main goal is to obtain such a derived curve in which no diagnostic information is lost. Choosing the right transformation method is key to the correct detection of pathology. With the right choice of the transformation method and the subsequent processing of the derived VCG, it can lead to a faster and more accurate diagnosis of a certain pathology. However, each transformation method has its advantages and disadvantages, especially in application to pathological records. In this case, it is important to know the effect of pathology on the ECG, because using the wrong transformation method may result in the loss of important information. For these reasons, the analysis was focused on records diagnosed with myocardial infarction, because the stages of the infarction affect both the QRS complex and the T wave. It was also confirmed by the cooperating cardiologist from the Ostrava Municipal Hospital that the diagnostic information of the derived VCG using the Kors regression method was preserved compared to the VCG measured by the Frank lead system and that application to current systems is feasible.

One of the most commonly used transformation methods is IDT and Kors regression method. More and more studies point to more accurate results based on the use of the Kors regression method [21], [34]–[37]. QLSV transformation is specialized mainly in a certain part of the ECG (QRS complex). The Quasi-orthogonal method is derived by approximation and is not suitable for further processing due to the high error rate.

The Quasi-orthogonal transformation is very rarely used due to the high error rate of this method [21], [38], [39]. Rubel *et al.* in [38] compared quasi-leads to another transformation matrix. The evaluation was done by calculating root mean square error (RMSE) and correlation. Rubel *et al.* in [38] confirmed that Quasi-orthogonal leads achieved the worst results.

Several publications have studied the transformation methods introduced by Kors. The authors in [36] and [35] discussed which of the available transformation methods will provide the QRS-T spatial angle most accurately to the angle obtained from Frank lead system. For their analysis, they used the two most commonly used transformation methods: Kors regression and IDT. The authors argue that the resulting values from the Kors regression method do not differ significantly from the values from Frank lead system. In their next study, the authors also focused on the QRS-T solid angle, but now in patients with hypertrophic cardiomyopathy. Similarly, Man *et al.* [34] analyzed the QRS-T angle from derived VCG records using the Kors regression method and IDT. In the comparison between the derived records and the records measured by the Frank lead system, it was Kors regression method that achieved more accurate results.

Statistical evaluation of individual transformation methods was performed by Jaros *et al.* [40]. In their study, they examined the accuracy of transformation methods used on 50 physiological records. In their analysis, they performed a Mann-Whitney nonparametric median test on calculated MSEs and correlation coefficients between derived and directly measured VCG records. From their results Kors regression transformation was statistically the most accurate for X and Y leads compared to other transformation methods. The difference between Kors regression transformation and the PLSV and QLSV methods was not statistically significant for Z lead. From the overall evaluation in all leads, Kors regression method was also the most accurate.

Certain analyzed transformation methods can then also be used in clinical practice, given that a 12-lead ECG is the most common method for analyzing electrical activity in the heart. The use of VCG in clinical practice is especially appropriate in applications of machine learning and other algorithms using advanced computational algorithms [41]–[44]. It was in this analysis that the higher sensitivity of the VCG was found compared to the conventional ECG. By extracting certain features, a higher success rate of the pathology can be achieved. The first use of transformation methods was made in ECG systems of Marquette Electronics, Inc. used in [8] and [23]. With more modern technologies, these methods can be applied in practice and thus provide physicians with other important information that could contribute to early diagnosis.

## VI. Conclusion

The accuracy of transformation methods is key to the correct interpretation of derived VCGs. If the transformation method is used correctly, it will increase the chance of successful pathology detection from VCG. In this work, a total of four transformation methods were analyzed: Kors regression, IDT, QLSV and Quasi-orthogonal method for possible use in clinical practice on 12 lead ECG data. These transformation methods were applied to 30 pathological records diagnosed with myocardial infarction from the PTB physionet database. The results of the transformation methods were compared with the original Frank lead system based on the MSE calculation. The MSE values for the individual leads (X, Y, Z) were the input values for the detailed statistical analysis, where we wanted to verify that the values of the Kors regression method achieve the lowest error rate in the signal transformation. The nonparametric multiselective Kruskal-Wallis test was used for statistical analysis, where we rejected the null hypothesis. To complete our statistical test, a post-hoc analysis using the Dunn test was required. Based on the results of the Dunn test, it was determined which transformation method can provide the most accurate derived orthogonal lead systems with respect to the directly measured VCG. The statistical analysis shows that, as a whole method, Kors regression method achieves the best values and can be considered the most accurate in application to the records diagnosed with myocardial infarction. The use of this linear transformation method in clinical systems can contribute to the extraction of spatial features for subsequent analysis and thus achieve a more accurate diagnosis.
